# A modified Sydney system for the diagnosis of chronic gastritis in dogs

**DOI:** 10.1186/s13028-020-00542-2

**Published:** 2020-08-12

**Authors:** Jolanta Spużak, Marcin Jankowski, Krzysztof Kubiak, Kamila Glińska-Suchocka, Rafał Ciaputa

**Affiliations:** 1 Department of Internal Medicine and Clinic of Diseases of Horses, Dogs and Cats, Faculty of Veterinary Medicine, Wroclaw University of Environmental and Life Sciences, pl. Grunwaldzki 47, 50-366 Wrocław, Poland; 2 Department of Pathology, Faculty of Veterinary Medicine, Wroclaw University of Environmental and Life Sciences, ul. C.K. Norwida 31, 50-375 Wrocław, Poland

**Keywords:** Dogs, Endoscopic examination, Gastritis, Histopathological examination, Sydney system

## Abstract

**Background:**

The Sydney system for assessing inflammatory lesions in the gastric mucosa is based on endoscopic and histological examinations. This study aimed to apply the Sydney system to diagnose gastritis in dogs. The study also compared the results of endoscopic and histological examinations conducted on gastric mucosal biopsy specimens. A total of 56 dogs with chronic vomiting were analyzed in the study. The physical appearance of the gastric mucosa was assessed through endoscopic examination, while the severity of the gastric inflammation, inflammation activity, glandular atrophy, and intestinal metaplasia were assessed by histopathological examination.

**Results:**

The endoscopic examination confirmed the presence of inflammatory lesions affecting the gastric corpus and pylorus in all the dogs, although the severity of these lesions differed between the individuals. Reflux gastritis was the most commonly observed gastric inflammation. In the histopathological examination of the gastric mucosal samples, inflammatory lesions were found in the gastric corpus of 53 dogs, while 55 dogs had lesions in the pylorus. This corresponds to a 96.4% agreement between the methods.

**Conclusions:**

The Sydney system is a useful tool for macroscopic and microscopic assessment of changes in the gastric mucosa as it enables the determination of inflammation type and severity, which helps the canine gastroenterologists to reliably compare the results of the tests performed in different facilities. Besides, the use of the Sydney system in diagnosing lesions facilitates the selection and effective monitoring of treatment. However, despite a high rate of agreement between the results of endoscopic and histopathological examinations, it is recommended to use both these methods for the assessment of the gastric mucosa in dogs.

## Background

Gastritis is one of the most common gastrointestinal diseases in dogs [1–4]. However, it is impracticable to unequivocally diagnose the disease and its cause based solely on medical history and clinical examination. Hence, gastroscopy should be deployed. This procedure indicates the character and extent of the lesions, while allowing to identify the sites for collecting gastric mucosal samples. For this reason, gastroscopy and histopathological examination of gastric mucosal samples are considered as the gold standard for the diagnosis of gastritis [2–10].

In veterinary medicine, at present, there are no specific morphological standards available for diagnosing the causes of gastritis and assessing its severity [2–6, 8, 10–13]. Therefore, many clinicians and veterinary pathologists consider the histopathological examination results of gastric mucosal samples to be controversial and inconclusive, which negatively affects the selection of treatment [5]. A study conducted by Willard et al. [13] showed that the agreement rate of diagnoses made by five independent histopathologists was only 50%.

Evaluating the correlation between the results of gastroscopic and histological examinations of gastric mucosal samples taken during endoscopy is of great significance [2, 3]. However, in many cases, it is difficult to establish such a correlation, possibly due to the lack of standard protocols for simultaneous macroscopic assessment of the gastric mucosa and histopathological examination of the mucosal samples [2, 3, 10].

In human medicine, the Sydney system is used for the assessment of inflammatory lesions in the stomach. This system was introduced at the 9th World Congress of Gastroenterology in 1990 [14–17] and updated in 1994 during the Houston session (Houston Gastritis Workshop) [17, 18]. As the classification system takes into account the results of endoscopic and histological examinations, it enables simultaneous macroscopic and microscopic assessment of the gastric mucosa [14–18]. The “endoscopic division” of the Sydney system includes the assessment of the topographical distribution of lesions (gastric corpus, antrum, the entire stomach) and the categories of endoscopic images, which may indicate erythematous/exudative gastritis, flat erosive gastritis, raised erosive gastritis, reflux gastritis, hemorrhagic gastritis, atrophic gastritis, and rugal hyperplastic gastritis [3, 14, 16]. The “histological division” of the system considers the etiological, topographical, and morphological features. This division includes the assessment of the severity of inflammation, inflammation activity, glandular atrophy, intestinal metaplasia, and *Helicobacter* spp. infection. The updated Sydney system classifies chronic gastritis into three categories: nonatrophic, atrophic, and special forms, i.e. chemical, granulomatous, eosinophilic, radiation-associated, lymphocytic, and infectious [3, 15, 16, 18–20].

In veterinary medicine, the guidelines developed by the World Small Animal Veterinary Association (WSAVA) Gastrointestinal Standardization Group are the only available standards for endoscopic and histopathological assessments of canine and feline gastritis. These guidelines enable the quantitative and qualitative assessment of macroscopic changes that are detected through endoscopy as well as the assessment of the quality and severity of microscopic changes in the gastric mucosa [5, 10]. However, these standards are not frequently applied by the canine and feline gastroenterologists, as demonstrated by a few publications on the subject that use them. The situation is further complicated by the fact that different gastroenterologists diagnose gastritis with the use of different classification systems.

As there is no common standard available for the quantitative and qualitative assessment of changes affecting the gastric mucosa in dogs with gastritis, we aimed to apply and evaluate the usefulness of the Sydney system in diagnosing gastritis in dogs, and compare the severity of lesions observed during endoscopic examination with those found through histopathological examination of the gastric mucosal samples.

## Methods

This retrospective study was conducted on 56 dogs of various breeds and both sexes (29 males and 27 females), aged 1–13 years (mean age 6 years). They were referred to the Endoscopic Laboratory of the Department of Internal Medicine and Clinic of Diseases of Horses, Dogs and Cats of Wroclaw University of Environmental and Life Sciences, Poland for gastroscopy due to chronic vomiting (lasting more than 3 weeks). The study neither included clinically healthy animals nor those with simultaneous symptoms of diarrhea and vomiting. In total, the dogs represented 27 breeds, while 11 were of mixed breed (Table 1).

**Table 1:** Signalment and clinical signs in the examined dogs

No.	Signalment			Clinical signs
	Breed	Age (years)	Sex	
1.	Labrador Retriever	7	Female	Vomiting of gastric contents
2.	Mixed breed	6	Female	Bilious vomiting, wheeze
3.	Mixed breed	9	Male	Vomiting of gastric contents, wheeze
4.	Boxer	5	Female	Bilious vomiting, salivation
5.	Shiba Inu	1	Male	Bilious vomiting, cough
6.	Mixed breed	3	Female	Hematemesis
7.	Mixed breed	10	Female	Bilious vomiting
8.	Shih Tzu	4	Female	Bilious vomiting, belching, stress
9.	Cocker Spaniel	5	Female	Bilious vomiting, frequent swallowing, stress
10.	Mixed breed	12	Male	Bilious vomiting, cough
11.	West Highland White Terrier	11	Female	Bilious vomiting, frequent swallowing, stress
12.	Mixed breed	7	Male	Bilious vomiting
13.	Samoyed	6	Male	Bilious vomiting, stress
14.	Irish Setter	13	Male	Bilious vomiting, frequent swallowing, eating grass, stress
15.	Labrador Retriever	9	Female	Bilious vomiting, salivation, eating grass, frequent swallowing, stress
16.	French Bulldog	1	Male	Bilious vomiting, stress
17.	Leonberger	6	Male	Vomiting of gastric contents, decrease in appetite, salivation
18.	Labrador Retriever	1	Female	Vomiting of gastric contents, cough
19.	Bichon Frise	2	Female	Bilious vomiting
20.	Scottish Terrier	13	Male	Bilious vomiting, abdominal pain
21.	Mixed breed	12	Male	Bilious vomiting
22.	German Shepherd	5	Female	Hematemesis
23.	Boxer	7	Male	Vomiting of gastric contents, frequent swallowing
24.	Bracco Italiano	1	Female	Bilious vomiting
25.	Bernese Mountain Dog	11	Male	Bilious vomiting, cough, frequent swallowing, salivation
26.	Afghan Hound	9	Male	Vomiting of gastric contents
27.	French Bulldog	9	Male	Bilious vomiting, salivation
28.	Mixed breed	5	Female	Bilious vomiting
29.	Yorkshire Terrier	7	Female	Bilious vomiting, stress
30.	Polish Hunting Dog	3	Male	Hematemesis
31.	Shih Tzu	12	Male	Bilious vomiting
32.	Yorkshire Terrier	4	Male	Hematemesis, lack of appetite
33.	German Shepherd	5	Female	Bilious vomiting, frequent swallowing, halitosis
34.	Border Collie	4	Male	Vomiting of gastric contents, abdominal pain, bubbling
35.	Rottweiler	7	Male	Bilious vomiting, salivation
36.	Labrador Retriever	12	Male	Bilious vomiting, cough
37.	Toy Poodle	5	Male	Bilious vomiting, salivation, frequent swallowing
38.	Shih Tzu	2	Female	Bilious vomiting
39.	Pug	4	Male	Bilious vomiting, cough
40.	Labrador Retriever	1	Male	Bilious vomiting, halitosis
41.	Beagle	8	Female	Bilious vomiting
42.	American Staffordshire Terrier	2	Female	Vomiting of gastric contents, cough, wheeze
43.	Mixed breed	12	Female	Bilious vomiting, frequent swallowing
44.	Cocker Spaniel	8	Female	Bilious vomiting
45.	Mixed breed	13	Female	Bilious vomiting
46.	Yorkshire Terrier	1	Female	Vomiting of gastric contents
47.	American Staffordshire Terrier	2	Female	Bilious vomiting, regurgitation
48.	Jack Rusell Terrier	4	Female	Bilious vomiting, eating grass
49.	Shih Tzu	4	Female	Bilious vomiting, frequent swallowing, salivation, stress
50.	French Bulldog	1	Male	Vomiting of gastric contents, frequent swallowing, regurgitation
51.	Boxer	1	Male	Vomiting of gastric contents
52.	Labrador Retriever	1	Male	Bilious vomiting
53.	Mixed breed	8	Female	Hematemesis
54.	Labrador Retriever	11	Male	Vomiting of gastric contents
55.	Yorkshire Terrier	5	Male	Bilious vomiting
56.	Maltese	2	Male	Bilious vomiting, regurgitation

The study procedure followed a routine protocol and did not require the approval of the local ethics committee. Informed consent was obtained from the dog owners.

The endoscopic examination of the dogs was carried out using the Olympus PCF-PH190I videoendoscope 24 h after they were abstained from food and 6 h after abstaining from water. Esophagogastroscopy was performed under general anesthesia. The dogs were premedicated with xylazine at a dose of 1 mg/kg body weight (bw) and atropine at 0.05 mg/kg bw, which were administered intramuscularly in a single injection. General anesthesia was induced with propofol at a dose of 4 mg/kg bw and maintained with the intravenous administration of this drug.

During the endoscopic examination, three samples of gastric mucosa were collected from the gastric corpus and three from the pylorus using Olympus FB-54U-1 endoscopic forceps. These biopsies were fixed in 7% neutral buffered formalin. Microscopic slides with mounted gastric mucosal samples were stained with hematoxylin and eosin.

The Sydney system was applied for the assessment of macroscopic and microscopic changes in the gastric mucosa [14–16, 18]. The severity of the endoscopic and histopathological changes was evaluated using a 4-point scale as follows: (0) no lesion, (1) mild lesions, (2) moderate lesions, and (3) severe lesions. The pathologist who assessed the samples was aware of the clinical condition of each dog.

Fisher’s exact test was used to calculate the difference in the incidences of different endoscopic image categories. The Mann–Whitney *U* test was performed to compare the severity of inflammatory lesions observed in the gastric corpus and pylorus during the endoscopic examination.

The Wilcoxon rank test was used to compare:

- The mean severity of inflammatory lesions observed during endoscopic examination in the gastric corpus and pylorus with inflammation activity determined through histopathological examination;
- The mean severity of inflammatory lesions observed during endoscopic examination in the gastric corpus and pylorus with inflammation intensity determined through histopathological examination;
- The mean value of inflammation activity in the gastric corpus and pylorus;
- The mean value of inflammation intensity in the gastric corpus and pylorus; and
- The severity of lesions observed during endoscopic examination (combined mean score from the gastric corpus and pylorus) and the severity of those observed in the histopathological examination (combined mean score from the gastric corpus and pylorus).

The proportion *Z*-test was conducted to compare the incidences of atrophy and metaplasia in the gastric corpus with those in the pylorus.

Statistical analyses were conducted with PQStat for Windows (version 1.6.2), produced by PQStat Software. A 5% level of significance was used in all the analyses.

## Results

**Fig. 1:**
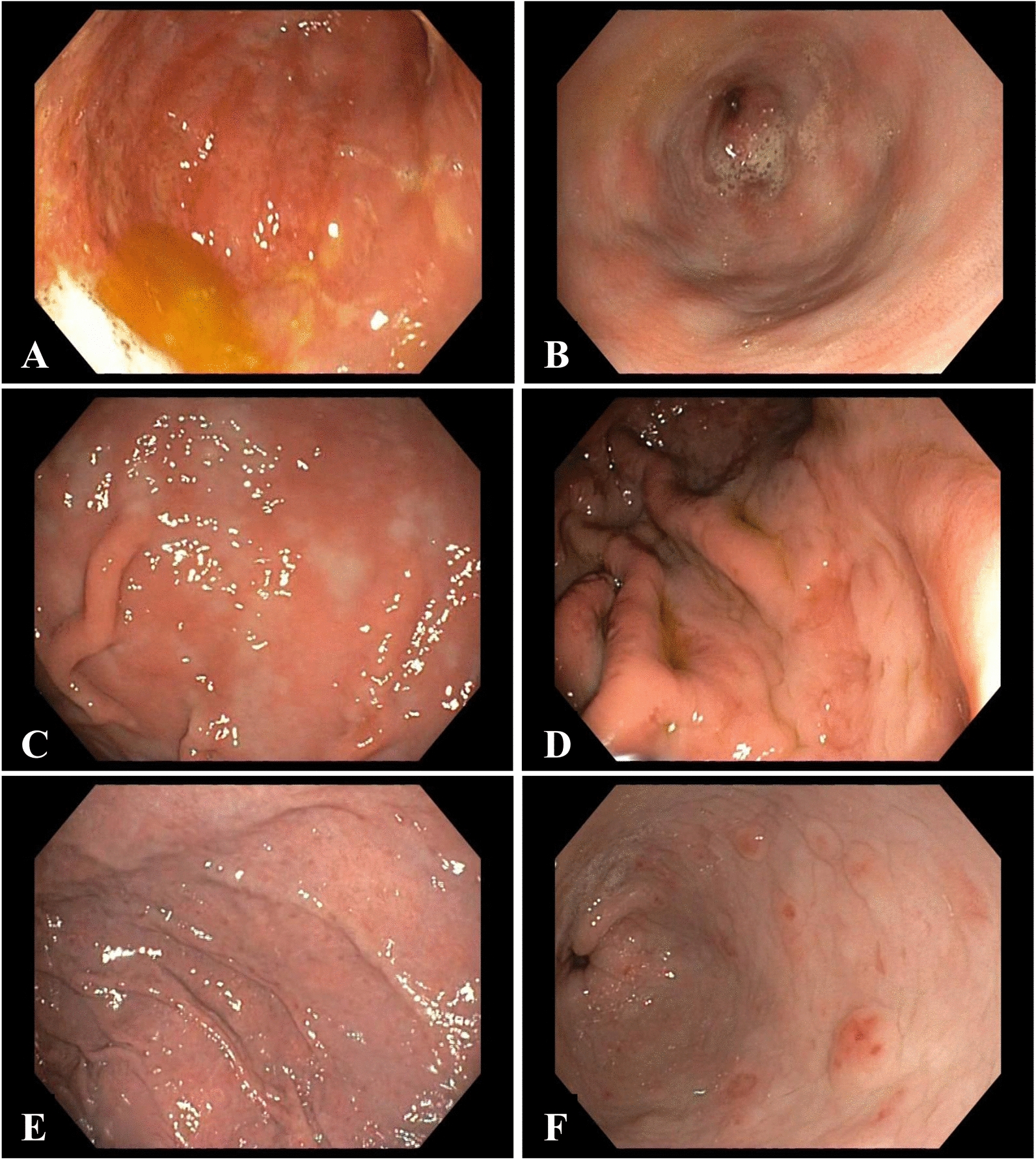
Endoscopic images of gastritis in dogs. **a**, **b** reflux gastritis, **c** erythematous gastritis, **d** hypertrophic gastritis, **e** gastritis with flat erosions, **f** gastritis with raised erosions

The endoscopic examination revealed the presence of inflammatory lesions within the gastric corpus and pylorus in all the examined dogs. The severity of the lesions differed between the individuals. In the mucosa of the gastric corpus, the severity of inflammatory lesions was diagnosed as mild in five cases (8.9%), moderate in 28 cases (50%), and severe in 23 cases (41.1%). In the pylorus, the mucosal lesions were found to be mild in four cases (7.1%), moderate in 30 cases (53.6%), and severe in 22 cases (39.3%). No statistically significant differences were found in the severity of inflammation between the gastric corpus and pylorus. Based on endoscopic imaging, reflux gastritis (Fig. 1a, b) was diagnosed in 34 cases (60.7%), erythematous gastritis (Fig. 1c) was diagnosed in 15 cases (26.8%), and hyperplastic gastritis (Fig. 1d) was diagnosed in four cases (7.1%). Erosive gastritis was detected in three cases (5.4%), of which flat erosions (Fig. 1e) were observed in two cases and raised erosions (Fig. 1f) were observed in one case. The difference in the incidences of the endoscopic image categories was found to be statistically significant (P < 0.001). For reflux gastritis, varying severity of mucosal redness was observed, in addition to the presence of bile in the stomach lumen. Furthermore, streaky reddening of the mucosa radiating from the pyloric sphincter was observed in the pylorus. Few erythematous lesions and a finely granulated mucosal surface were observed in erythematous gastritis. For erosive gastritis, reddening of the gastric mucosa was detected along with lesions that ran along the folds of the mucous membrane. In hyperplastic gastritis, thickened folds that were resistant to insufflation were observed in the stomach.

During the histopathological examination of the gastric mucosal samples, lesions were observed in the gastric corpus in 53 dogs (94.6%) and pylorus in 55 dogs (98.2%). Differences between the histopathological and endoscopic examinations in the diagnosis of inflammatory lesions in the gastric corpus were evident in three dogs (5.36%), and in the case of the gastric pylorus, in one dog (1.79%). In all, the differences in the diagnosis of inflammatory changes with the histopathological and endoscopic examination of the gastric corpus and pylorus amounted to 3.6%.

Based on the histopathological examination of the gastric mucosal samples, lymphoplasmacytic inflammation was diagnosed in all the examined dogs.

**Fig. 2:**
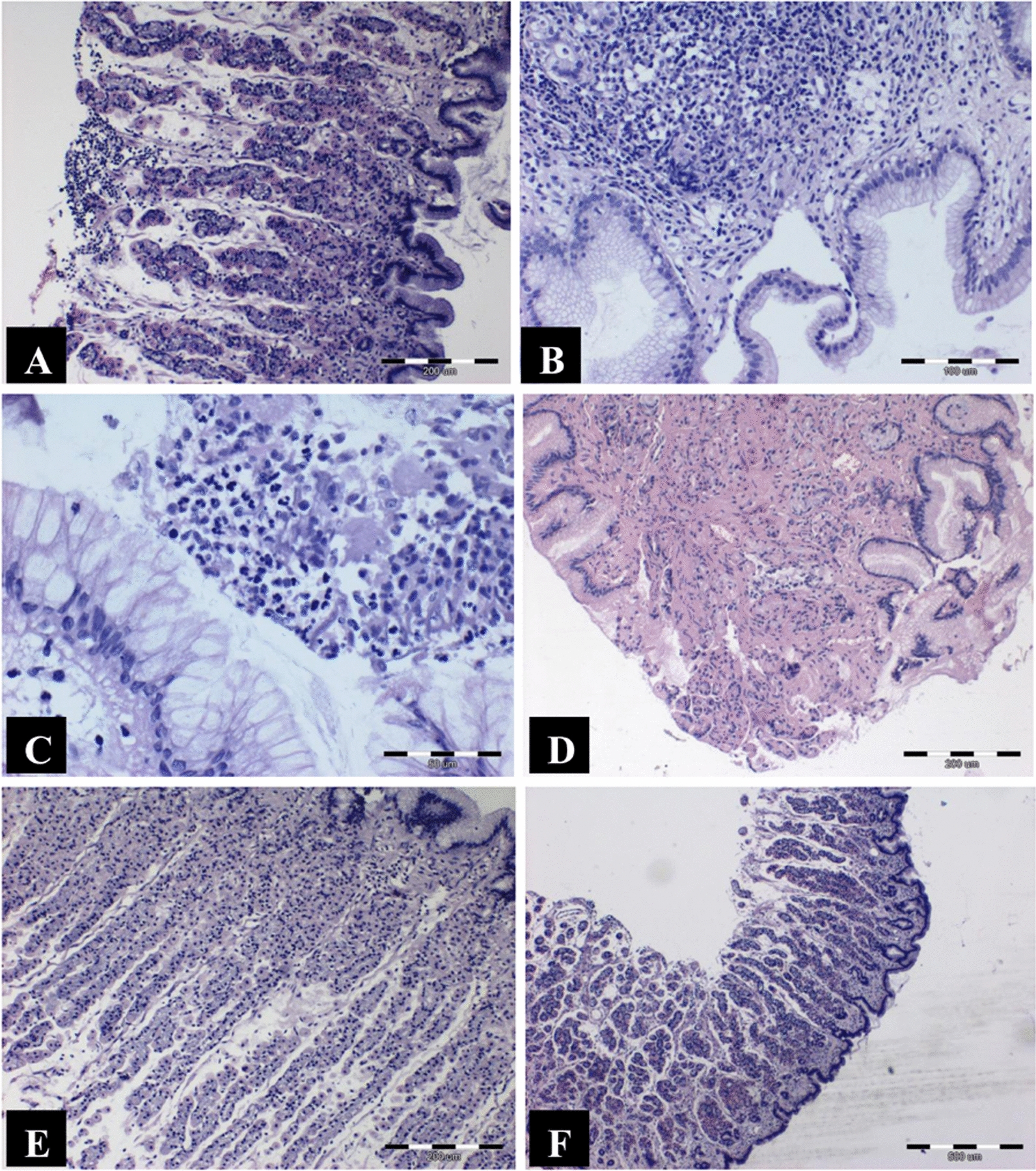
Photomicrographs of gastric mucosa in dogs: hematoxylin and eosin staining. **a**, **b** infiltration of lymphocytes in the gastric mucosa (inflammation intensity) (Obj. A × 10, B × 20). **c** infiltration of granulocytic cells in the gastric mucosa (inflammation activity) (Obj.  ×40). **d** glandular atrophy (Obj.  ×10). **e**, **f** intestinal metaplasia (Obj. E  ×10, F  ×4)

In the gastric corpus, the intensity of inflammatory changes was detected as mild in 25 cases (44.6%), moderate in 18 cases (32.1%), and severe in 10 cases (17.9%). No lesions were found in three cases (5.4%). In two of those cases, reflux gastritis was diagnosed through endoscopic examination, while erythematous gastritis was diagnosed in one case. The intensity of inflammatory lesions in the pylorus was rated as mild in 20 cases (35.7%), moderate in 21 cases (37.5%), and severe in 14 cases (25%) (Fig. 2a, b). No lesions were observed in one dog (1.8%), in which erythematous gastritis was diagnosed through endoscopic examination. However, no significant difference was observed for inflammation intensity between the gastric corpus and pylorus.

Active gastritis of the gastric corpus was diagnosed in 23 cases (41.1%), of which mild gastritis was found in 14 cases (25%) and moderate gastritis in nine cases (16.1%). Active gastritis of the pylorus was diagnosed in 25 cases (44.6%), of which 14 cases were mild (25%), seven were moderate (12.5%), and four were severe (7.1%) (Fig. 2c). However, no significant differences were found between inflammation activity in the gastric corpus and pylorus.

Glandular atrophy of the gastric corpus was observed in eight cases, of which mild atrophy was seen in four cases (7.1%), moderate atrophy in three cases (5.4%), and severe atrophy in one case (1.8%). Glandular atrophy of the pylorus was seen in 23 cases (41.1%), of which nine cases (16.1%) showed mild atrophy, 12 cases (21.4%) showed moderate atrophy, and two cases (3.6%) showed severe atrophy (Fig. 2d). A significant difference (P = 0.003) was found in the incidence of glandular atrophy between the gastric corpus and the pylorus.

The following changes were observed through endoscopic examination in dogs that had histologically confirmed glandular atrophy in the gastric corpus: erythematous gastritis in four, reflux gastritis in three, and erosive gastritis in one. The following types of inflammation were observed in dogs that had histologically confirmed glandular atrophy in the pylorus: reflux gastritis in 14, erythematous gastritis in eight, and erosive gastritis in one.

Moderate intestinal metaplasia was found in the gastric corpus in one dog (1.8%). Intestinal metaplasia was found in the pylorus in five cases (8.9%). Of these, two (3.6%) had mild metaplasia, two (3.6%) moderate, and one (1.8%) suffered from severe metaplasia (Fig. 2e, f). However, there were no significant differences observed in the incidence of intestinal metaplasia in the gastric corpus and pylorus.

In one dog, histopathological examination indicated the diagnosis of intestinal metaplasia in the gastric corpus, whereas endoscopic examination resulted in the diagnosis of reflux gastritis. In dogs in which pyloric intestinal metaplasia was confirmed through histology, the endoscopic examination indicated the diagnosis of reflux gastritis in four cases and erythematous gastritis in one case (Table 2).

**Table 2:** Endoscopic diagnosis and histopathological changes in the examined dogs

Sydney system										
Endoscopic diagnosis		Histopathological diagnosis								
		Corpus					Pylorus			
	n	Scale	I	A	GA	IM	I	A	GA	IM
			n	n	n	n	n	n	n	n
Reflux gastritis	34	0	2	19	32	33	0	19	21	30
		1	16	9	1	0	12	8	4	1
		2	10	6	1	1	12	5	7	2
		3	6	0	0	0	10	2	2	1
Erythematous gastritis	15	0	1	10	12	15	1	9	7	14
		1	7	4	2	0	4	3	4	1
		2	4	1	1	0	6	2	4	0
		3	3	0	0	0	4	1	0	0
Hyperplastic gastritis	4	0	0	2	2	4	0	2	3	4
		1	1	1	1	0	2	2	0	0
		2	2	1	0	0	2	0	1	0
		3	1	0	1	0	0	0	0	0
Erosive gastritis	3	0	0	2	2	3	0	1	2	3
		1	1	0	0	0	2	1	1	0
		2	2	1	1	0	1	0	0	0
		3	0	0	0	0	0	1	0	0

Scale: 0: no lesions, 1: mild lesions, 2: moderate lesions, and 3: severe lesions

I, intensity; A, activity; GA, glandular atrophy; IM, intestinal metaplasia

The statistical analysis included a comparison of the intensity of inflammatory lesions observed through endoscopy with the intensity of inflammatory changes (infiltration of mononuclear cells) observed in the histopathological examination of the gastric mucosal samples. This comparison was carried out separately for the gastric corpus and pylorus. A significant difference was noted between the two diagnostic methods in the intensity of inflammation in the gastric corpus (P < 0.001) versus the pylorus (P < 0.001). Furthermore, the intensity of inflammatory lesions observed in the endoscopic examination of the gastric corpus and pylorus was compared with the activity of inflammatory changes (infiltration of neutrophils) observed through histopathological examination of the gastric mucosal samples. The gastric corpus and pylorus were also assessed independently. A significant difference was noted between the two diagnostic methods in the intensity of inflammation in the gastric corpus (P < 0.001) and pylorus (P < 0.001).

**Fig. 3:**
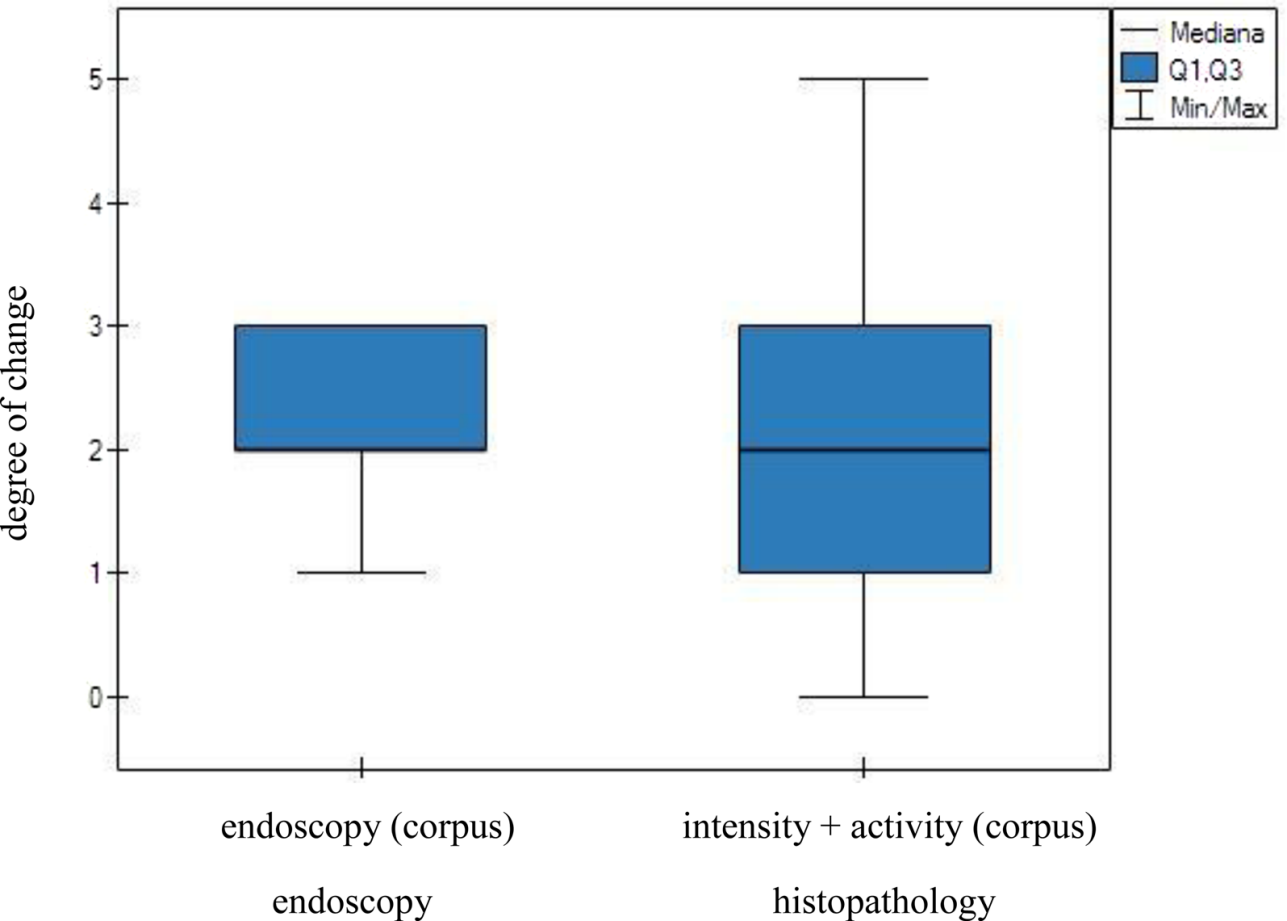
Endoscopic and histological analyses of the severity of inflammatory lesions in the gastric corpus. No statistically significant differences were observed between the two diagnostic methods

**Fig. 4:**
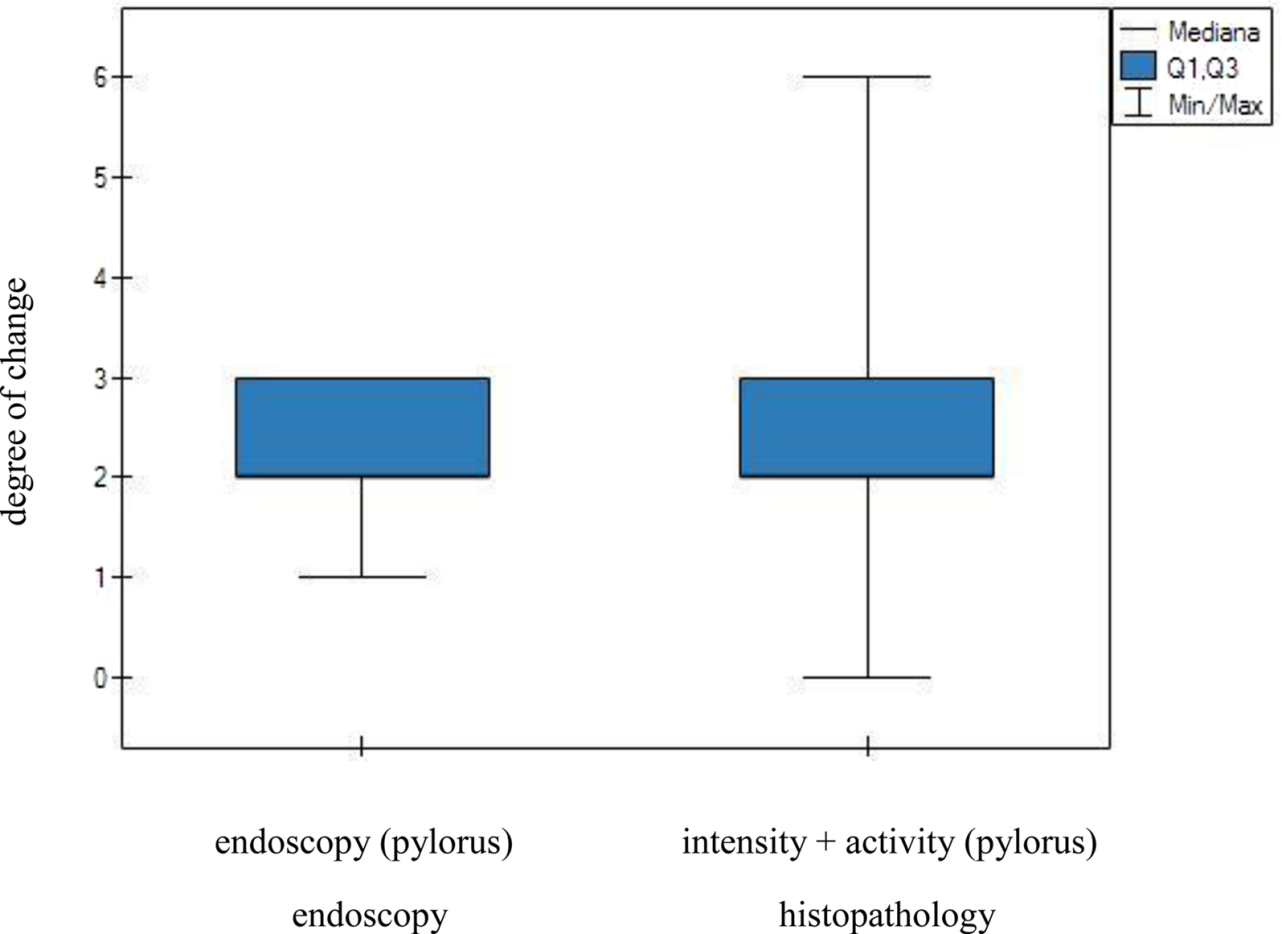
Endoscopic and histological analyses of the severity of inflammatory lesions in the pylorus. No statistically significant differences were observed between the two diagnostic methods

The intensity of lesions observed through endoscopic examination of the gastric corpus was then compared with both the intensity and activity of inflammation as assessed by the histopathological examination of the gastric mucosal samples. However, this comparison revealed no significant differences between the two techniques (Fig. 3). Similarly, an analogous analysis performed for the pylorus revealed no significant differences between the techniques (Fig. 4).

**Fig. 5:**
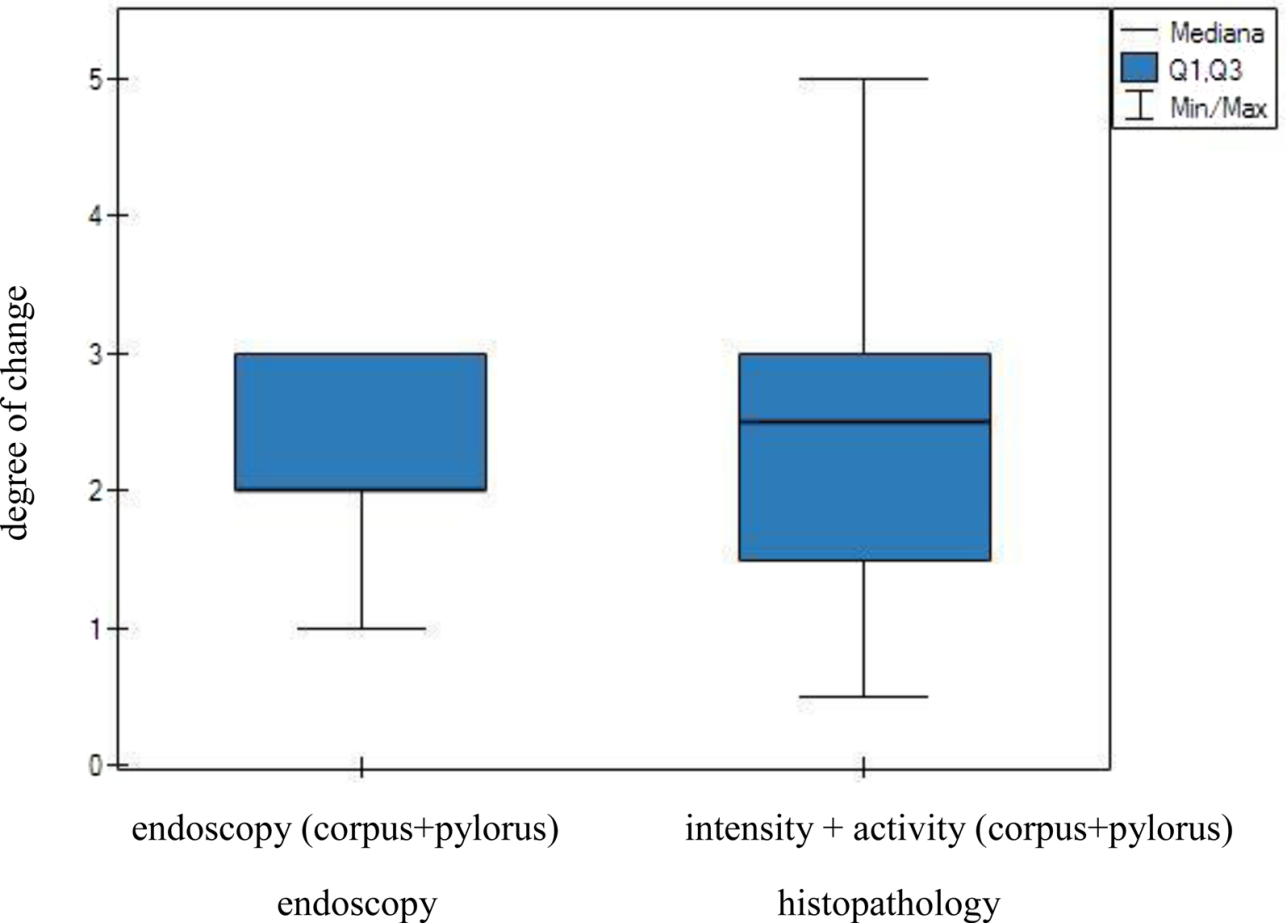
Endoscopic and histological analyses of the severity of inflammation in the gastric corpus and pylorus. No statistically significant differences were observed between the two diagnostic methods

Besides, lesions within the gastric corpus and pylorus found in the endoscopic examination were compared with the lesions found in the histopathological examination of the two regions. The mean values of the endoscopic examination of the gastric corpus and pylorus were calculated. While calculating the mean values of the histopathological examination of the gastric corpus and pylorus, included both the intensity and activity of the inflammation were taken into account. The statistical analysis showed no significant differences between the results of endoscopic and histopathological examinations (Fig. 5).

## Discussion

Gastroscopic and histopathological examinations of samples of gastric mucosa taken during endoscopy are the two most widely used tools to detect gastritis in dogs and cats [2–6, 8, 10].

A few studies in veterinary medicine have outlined the criteria for the endoscopic assessment of gastric inflammatory lesions in dogs [2, 5, 10], while only one study has used the endoscopic division of the Sydney system [3]. Since the Sydney system classifies inflammatory changes based on their character and location in the stomach, it is easier for clinicians to interpret the changes [3, 14, 16, 18]. In this study, reflux gastritis was the most frequently diagnosed gastric inflammation in the examined dogs. The inflammatory changes affected the entire stomach in all the dogs, and no differences were observed in the severity of inflammatory lesions between the gastric corpus and pylorus. Most of the lesions were moderate or severe (92%). Colakoğlu et al. [2] and Ülgen et al. [12] thoroughly examined the endoscopic images in their study to determine the type of inflammatory changes diagnosed in dogs utilizing gastroscopy, but they neither considered the location and severity of the lesions in the individual parts of the stomach nor assigned them to any specific category. Marchesi et al. [8] assessed macroscopic lesions in the stomach by dividing them into acute inflammation, chronic inflammation, and nodular gastropathy. The present study indicates that the endoscopic division of the Sydney system is a superior tool to assess macroscopic lesions in the gastric mucosa as it enables an accurate description of the type of mucosal change, its extent, and severity.

In this study, the application of the histological division of the Sydney system helped in assessing the type of gastritis and its severity. Our study revealed lymphoplasmacytic gastritis in all the dogs, which is concurrent with other studies, indicating that this type of inflammation in dogs is most common [1, 2, 4, 6, 10]. In the majority of dogs, the inflammatory changes affected both the corpus and the pyloric part of the stomach (92.9%). However, the severity of lesions may differ depending on the area of the stomach [3]. In our study, glandular atrophy and intestinal metaplasia were found more often in the pylorus than in the gastric corpus. This observation is also confirmed by Barwijuk-Machała et al. in their study on humans [21]. However, glandular atrophy and intestinal metaplasia do not always accompany inflammatory lesions, even the severe ones [3]. In our opinion, a more frequent occurrence of glandular atrophy and intestinal metaplasia is associated with a higher incidence of duodenogastric reflux in dogs. Thus, determining the presence and severity of glandular atrophy and intestinal metaplasia is clinically significant, as these changes may influence the choice of treatment and patient prognosis. This diagnosis is particularly critical for intestinal metaplasia, which is considered as the early stage of gastric neoplasia, such as gastric adenocarcinoma [4, 22–24].

Although extensive research has been conducted on the correlation between macroscopic and histopathological changes of the gastric mucosa, its existence is still highly controversial. In human medicine, the agreement between macroscopic and microscopic assessments of gastric mucosal lesions reported to range from 54 to 97% [14, 15, 17, 18, 24–32]. However, in veterinary medicine, very few studies have compared the agreement between the incidence of endoscopic lesions and the histopathological examination of samples taken from the canine stomach [2, 8]. In our study, the agreement between the diagnosis of inflammatory lesions in the gastric mucosa based on endoscopic and histopathological examinations was 96.4%. The severity of lesions diagnosed through the endoscopic examination corresponded with that observed through the histopathological examination if the latter included the intensity and activity of inflammation.

Colakoğlu et al. [2] used the WSAVA standards to perform microscopic and macroscopic assessments of the gastric mucosa and found that the correlation between these two procedures was 71.8%. Marchesi et al. [8] found a correlation of 75.4% between the two procedures, although the authors did not mention the standard on which they based their histopathological assessment. The difference between our results and those of other authors concerning the correlation between endoscopic and histopathological examinations may be influenced by the following factors: (1) the experience of the clinician performing the endoscopic study, (2) the number of the collected biopsy samples of the gastric mucosa, (3) the quality of the gastric mucosal samples, (4) the type of sample preparation before the histopathological assessment, and (5) the standard used to assess endoscopic and histopathological lesions in the stomach. Similar factors have also been proposed by other authors [6, 7, 10, 13, 33].

Based on the obtained results, the authors believe that despite the high rate of agreement between the results of the endoscopic assessment of the gastric mucosa and the morphological examination of the mucosal samples, the histopathological examination should always be considered while diagnosing gastrointestinal disorders in dogs. This is because a histopathological examination not only determines the inflammation type and severity but also reveals the other changes within the stomach, such as glandular atrophy, intestinal metaplasia, or neoplastic lesions. These findings have been proved by Ajayi et al. [25] in their study on humans and by Marchesi et al. [8] in their study on dogs.

## Conclusions

The Sydney system is a useful tool for the macroscopic and microscopic assessment of changes in the gastric mucosa as it enables the determination of inflammation type and severity. Moreover, this system allows the canine gastroenterologists to reliably compare the results of tests performed in different facilities. Besides, the diagnosis of lesions following the Sydney system facilitates the selection and effective monitoring of treatment. However, despite a high correlation between the results of endoscopic and histopathological examinations, it is recommended to use both methods for examining the gastric mucosa in dogs.

## Data Availability

The data sets analyzed during the current study can be availed from the corresponding author on a reasonable request.

## Abbreviation

WSAVA: World Small Animal Veterinary Association
